# Characteristics of Patients with Persistent COVID-19 Symptoms and Unscheduled Return Visits to a Centre for COVID-19 Evaluation

**DOI:** 10.3390/diseases12090199

**Published:** 2024-08-30

**Authors:** Silvia Nica, Remus Iulian Nica, Horia Alexandru Nica, Daniela Miricescu, Mohamed Abuzied Ali Khattab Abdelfatah, Oana Maria Schiopu, Ioan Cristian Nedelcu, Danut Gheorghe Cimponeriu, Constantin Stefani, Iulia-Ioana Stanescu-Spinu, Mariana Cătălina Ciornei

**Affiliations:** 1 Emergency Discipline, University Hospital of Bucharest, 050098 Bucharest, Romania; silvia.nica@umfcd.ro (S.N.); catalina.ciornei@umfcd.ro (M.C.C.); 2 Department of Emergency and First Aid, Carol Davila University of Medicine and Pharmacy, 050474 Bucharest, Romania; 3 Central Military Emergency University Hospital “Dr. Carol Davila”, 010825 Bucharest, Romania; moeran2003@yahoo.com (M.A.A.K.A.); lea13ael@yahoo.com (O.M.S.); cristian.nedelcu@umfcd.ro (I.C.N.); 4 Discipline of General Surgery, Faculty of Midwifery and Nursing, Carol Davila University of Medicine and Pharmacy, 050474 Bucharest, Romania; 5 Faculty of Medicine, Carol Davila University of Medicine and Pharmacy, 8 Eroii Sanitari Blvd., 050474 Bucharest, Romania; horiaalexandru.nica2023@stud.umfcd.ro; 6 Discipline of Biochemistry, Faculty of Dentistry, Carol Davila University of Medicine and Pharmacy, 050474 Bucharest, Romania; 7 Discipline of Genetics, Faculty of Biology, University of Bucharest, 010041 Bucharest, Romania; danut.cimponeriu@bio.unibuc.ro; 8 Department I of Family Medicine and Clinical Base, “Dr. Carol Davila” Central Military Emergency University Hospital, 051075 Bucharest, Romania; constantin.stefani@umfcd.ro; 9 Discipline of Physiology, Faculty of Dentistry, Carol Davila University of Medicine and Pharmacy, 8 Eroii Sanitari Blvd, 050474 Bucharest, Romania; iulia.stanescu@umfcd.ro; 10 Discipline of Physiology, Faculty of Medicine, Carol Davila University of Medicine and Pharmacy, 050474 Bucharest, Romania

**Keywords:** COVID, COVID centre, chronic diseases, vaccination, age

## Abstract

**Background:** This retrospective study aimed to evaluate the characteristics of patients with long COVID syndrome. **Methods:** This study included 457 adults who had at least one persistent symptom after COVID-19 infection. **Results:** The median time interval between the last SARS-CoV-2 infection and emergency room presentation was 3 months. Older patients had comorbidities (61.7 vs. 44.9 years, *p* < 0.0001), moderate or severe forms of COVID-19 (61.2 vs. 50.9 years, *p* < 0.0001), and respiratory symptoms (56.1 vs. 52.0 years, *p* = 0.0027). Non-vaccinated patients were older than vaccinated patients (56.0 vs. 51.5 years, *p* = 0.0008) and had residual lung abnormalities following COVID-19 infection (51.5% vs. 36.8%, *p* < 0.003). The time interval between the last SARS-CoV-2 infection and the hospital evaluation was shorter for vaccinated patients (3.2 vs. 3.9 months, *p* < 0.0001) and those with mild forms (3.3 vs. 4.12 months, *p* = 0.0001) versus non-vaccinated individuals. After the last SARS-CoV-2 infection, 107 patients developed impaired fasting glucose, impaired glucose tolerance, or diabetes mellitus, being patients with already known chronic diseases (*p* = 0.0002), or hypertension (*p* = 0.001). **Conclusions:** Our study pointed out the heterogeneity of symptoms following COVID-19, and they are associated with age, vaccination status, or severity of SARS-CoV-2 infection.

## 1. Introduction

COVID-19, caused by the SARS-CoV-2 virus [1], is responsible for the latest pandemic SARS-CoV-2 enters the cells using the angiotensin-converting enzyme 2 (ACE2) [2] to bind with its Spike protein receptor. The spike protein contains the receptor-binding domain (RBD), which attaches to the ACE2 receptor by its six amino acid residues [3]. To complete entry into the cell, SARS-CoV-2 uses the active protease TMPRSS2 to ensure the link between spike protein and ACE2 [4]. Further, the infection induces a complex immune response associated with the synthesis of interleukins IL-6, IL-1β, IL-1α, IL-2, IL-7, IL-8, tumor necrosis factor (TNF-α), interferon-γ (IFN-γ), and C-reactive protein (CRP) [5,6]. Their release causes a cytokine storm, which leads to taste bud stem cell apoptosis, disrupting their renewal and thus affecting the taste function [7]. The majority (~80%) of the affected individuals develop mild to moderate forms of the disease, but some patients (5%) develop a critical or even fatal form of illness [1,8]. An acute COVID-19 episode lasts around five weeks, during which viral infection disables host cellular machinery, leading to cell death. These molecular events will lead to systemic hyperinflammation, followed by various injuries such as thrombosis, multi-organ failure, and even death [9]. ACE2 receptors are found in the epithelia of the respiratory, cardiovascular, and urogenital systems, gastrointestinal tract, liver, gallbladder, and nervous system [10]. The severity of this virus infection is closely related to the ACE2 capacity for binding and maturity [2]. In 70–80% of coronavirus-infected patients, it was reported that severe respiratory distress was observed by radiologic examination [11]. Regarding cardiac symptoms after COVID-19 infection, chest pain, palpitations, and postural tachycardia syndrome were recorded in patients after discharge from the hospital after an acute episode of infection [12]. Pre-existing health pathologies, such as cardiovascular diseases (CVDs), diabetes, obesity, liver and kidney damage, cancer, vitamin D deficiency, Epstein–Barr virus, and Parkinson’s disease, may enhance COVID-19 severity in both young and older populations [13,14]. Usually, this viral infection has a worse evolution when older age, male sex, and race (particularly black, Hispanic, and South Asian) are associated with various pre-existing pathologies [15]. Studies have shown that ongoing symptoms after the COVID-19 infection were more frequently reported in hospitalised patients than non-hospitalised patients, who reported only fatigue and cognitive impairment [16]. Interestingly, in patients who experienced long COVID-19 syndrome, neurological, neuropsychiatric, cardiopulmonary, gastrointestinal, and other complications (primary rheumatological complications) were significantly more often observed in female than in male patients [17,18,19,20].

SARS-CoV-2 may cause a post-acute syndrome with heterogeneous manifestations [21]. Long-term COVID-19 can affect a wide range of patients, from those with mild/moderate issues to those with severe forms, and involves various organs and systems such as the respiratory, cardiovascular, gastrointestinal, musculoskeletal, and neurological systems. Moreover, between 10 and 65% of patients who had mild or moderate COVID-19 present post-COVID-19 syndrome, characterised by fatigue, anxiety, depression, dyspnea, and impaired attention, concentration, and sleep [22,23,24,25,26]. Patients with persistent cognitive impairment syndrome, also called COVID-19 brain fog, reported perturbations regarding attention, memory, concentration, executive function, and information processing speed. Neuroinflammation is responsible for this situation, causing glial and neuronal cell dysregulation [27].

The risk of developing severe or fatal forms of the disease or persistent symptoms was significantly reduced by immunization with COVID-19 vaccines [17,28,29]. The high cumulative prevalence (9–63%) of long COVID has placed more than 100 million people in front of healthcare professionals [17,18,19,20]. The increased number of patients who complained of a decrease in the quality of life after SARS-CoV-2 infection and the large number of unscheduled return visits or repeat consultations led to the opening of the Centre for Evaluation of COVID-19 in the Carol Davila Military Emergency Hospital (CEC-CD) at the beginning of December 2021.

This retrospective study evaluated the characteristics of patients with persistent COVID-19 symptoms and unscheduled visits to the CEC-CD.

## 2. Materials and Methods

### 2.1. Medical Records

In this retrospective study, we evaluated medical records for 457 adults with at least one persisting symptom after COVID-19 and unscheduled visits to the CEC-CD between December 2021–June 2022, after the approval of the ethics committee of the CEC-CD (No. 620/09.08.2023). The methodology of this study is in accordance with the Helsinki Declaration.

The records refer to the different clinical characteristics of the patients, including those related to COVID-19 disease (e.g., disease severity, treatment, vaccination status). The most important persisting symptoms with onset during or after SARS-CoV-2 infection (e.g., fatigue, dyspnea, coughing, and headache, consisting of palpitations, chest pain, allergic reactions, and muscle and joint pain) were considered based on self-report. The data regarding myocarditis (based on angina, dyspnea, fatigue, elevated high sensitivity Troponin I-hs-TnI values, and suggestive echocardiography) and cardiovascular symptoms (e.g., palpitations, precordial pains, dyspnea), the presence of inflammatory syndrome (based on CRP values, increased fibrinogen levels, and erythrocyte sedimentation rate-ESR values), and residual lung involvement (identified by conventional radiology or CT) were also evaluated. Hs-TnI was determined by chemiluminescence assay, CRP using an automatic analyzer, ESR on an automatic reader, and fibrinogen by the Clauss method. The dataset used for this study did not include data from patients with mental illnesses, chronic dyspnea, or fatigue before COVID-19; an incomplete immunization schedule (e.g., one dose of vaccine from BioNTech (Mainz, Germany) or MODERNA (Cambridge, MA, USA)); or vaccination less than 2 weeks before infection with COVID-19.

### 2.2. Statistical Analysis

Statistical analysis was primarily performed by using RStudio for Windows. The Student’s *t*-test or Mann–Whitney U-test were used to compare the groups of data. The age of patients or the interval between the last SARS-CoV-2 infection and unscheduled return visits to the CEC-CD that exceed the mean value ± 3 standard deviations were considered outliers and were excluded from the analysis. The Bonferroni correction was used to adjust probability (*p*) values for 16 variables, and we considered the differences to be statistically significant if *p*< 0.003.

## 3. Results

This dataset includes medical records for adults who had at least one persisting symptom after COVID-19 which caused an unscheduled visit to the CEC-CD (Table 1).

**Figure 1:**
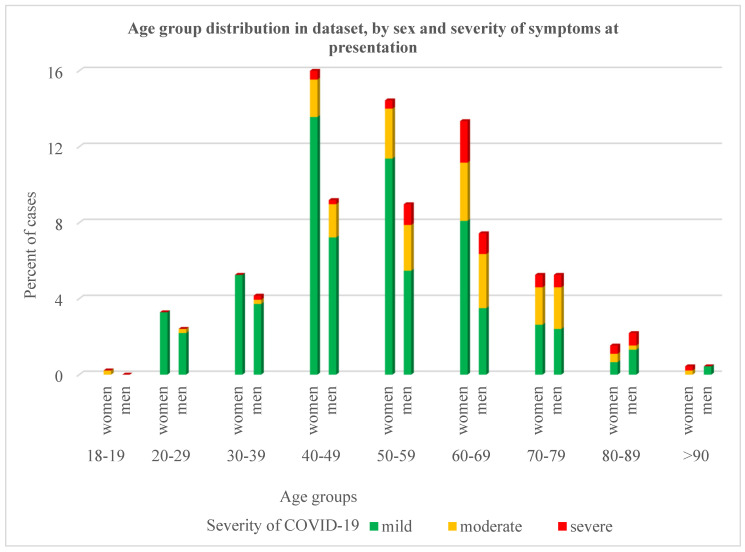
The distribution of cases from the dataset by age group, gender, and severity of symptoms at presentation to the CEC-CD.

The mean age of patients at presentation in the CEC-CD was 53.85 (range 18–91) years old; most of them belonged to the age group 40–69 years (73.4% of women; 63.9% of men) and had mild clinical COVID-19 symptoms (Figure 1).

The age at presentation was similar in both genders (*p* > 0.05) and in the subgroup of subjects under (women vs. men: 50.06 ± 11.58 vs. 49.3± 11.7 years old) or over (women vs. men: 76.67 ± 5.18 vs. 76.86 ± 6.32) the age of 70 years old. However, age presented significant differences between different subgroups of subjects. Patients who had comorbidities, moderate or severe forms of COVID-19, and respiratory symptoms (dyspnea, cough) were older compared to those who did not have these characteristics (Table 2).

The age of patients who had myocarditis (58.0 vs. 53.4 years old) or inflammatory syndrome (57.2 vs. 53.1 years old) tends to be higher (*p* = 0.02) than in those without these syndromes. Lung damage was present in 92 males and 111 females (44.42% of patients) from our dataset; 50.2% of them (43 males and 59 females) reported respiratory problems at the moment of presentation at the CEC-CD. Patients with post-COVID residual pulmonary involvement were older than those without such manifestations (58.78 vs. 49.91 years, *p*< 0.0001); they were more frequently identified in the non-vaccinated than in the vaccinated lot (51.48% vs. 36.82%, *p* = 0.002). An X-ray or CT scan performed during evaluation in the CEC-CD revealed that patients who had moderate forms of COVID-19 have different degrees of pulmonary fibrosis, whereas those with severe forms of SARS-CoV-2 infection showed significant pulmonary fibrosis, which may be associated with persistent ground-glass opacities. At least two signs or symptoms at the CEC-CD presentation were reported more frequently by patients who had chronic diseases compared to those without medical history (59.02% vs. 42.72%, *p* = 0.0005). A different distribution of medical history was identified in patients stratified according to the clinical severity of COVID-19 (Table 3).

Atopies were more frequently reported by women than by men (30.29% vs. 15.85%, *p* = 0.0004). The symptoms noted consisted of significant oculo-nasal catarrh and skin erythema in individuals without an allergic history. The age of patients with such symptoms seems lower compared to those who denied the existence of these manifestations (51.40 vs. 54.64 years old, *p* < 0.05).

The median time interval between the last SARS-CoV-2 infection and the CEC-CD presentation was 3 months (interval 1–15 months). For sixteen patients, this interval was considered an outlier. The distribution of remaining values was different in subjects stratified according to vaccination status, hypertension, the clinical form of COVID-19, or the treatment administered (Table 4).

Vaccinated patients, compared with those unvaccinated, were significantly younger (51.5 vs. 56.0 years, *p* < 0.001), had a mild form of COVID-19 (77.72% vs. 64.97%, *p* = 0.003), and had a shorter time interval between clinical onset of this disease to unscheduled visits to the CEC-CD (3.2 vs. 3.9 months, *p* < 0.0001). This time interval was also shorter in patients who had mild forms of COVID-19 compared to those with more severe forms of the disease (3.31 vs. 4.12 months, *p* = 0.0001).

Fifty patients were diagnosed with hypertension after COVID-19. They represent 22.22% of the patients with previous CVDs and 7.45% from the sublot of patients without these health problems (*p* < 0.0001). Patients with new-onset diabetes mellitus were more frequently detected in the subgroup of patients who had comorbidities (30.33% vs. 15.49%, *p* < 0.0002) or hypertension (42% vs. 21.13%, *p* = 0.001).

## 4. Discussion

There was estimated to be a significant number of cases of long COVID in Romania (2020: 79,500 cases; 2021: 415,000) [30]. The Romanian Ministry of Health decided in December 2021 to establish health assessment centres for patients with COVID-19 to relieve Emergency Units. In the CEC-CD, most patients were evaluated in the first six months after establishment, after which patient presentation decreased significantly. This observation shows why the evaluated dataset refers to patients who had unscheduled visits to the CEC-CD between December 2021–June 2022.

In a meta-analysis report, it was noted that 80% of the patients with a confirmed diagnosis of COVID-19 continued to have at least one overall effect beyond 2 weeks following acute infection [31]. A relevant physical and functional recovery during the first year after COVID-19 infection was observed for a significant percentage of patients, although in some patients, symptoms persisted for a longer period [32,33,34]. It was estimated that the post-COVID symptoms affect 7.8–35% of COVID-19 patients (this figure may be even higher in some particular groups of patients) [35,36,37,38].

We retrospectively investigated the post-COVID-19 persistent symptoms of 457 patients with mild or moderate general conditions who had unscheduled visits to the CEC-CD. Our results, like other previously published data, reveal that some manifestations of post-COVID syndrome such as cardio-vascular involvement) develop more frequently in women [39,40]. The risk of developing long COVID was estimated to be several times lower in patients in their twenties than in people in their sixties (1–2% vs. 5%) [41]. Generally, it is considered that older patients with COVID-19 (or at least until 70 years of age) have a higher risk of long-term COVID-19 and are prone to worse outcomes than younger patients [37,42,43,44]. In our study, 69 patients were at least 70 years old (36 were male; 41 were unvaccinated). Gender was not associated with statistically significant differences between subgroups of patients aged <70 or ≥70 years. However, the age of vaccinated patients is significantly lower compared to the age of unvaccinated subjects (51.5 vs. 56.03, *p* < 0.001).

The consensus WHO definition mentions that post-COVID-19 occurs usually 3 months from the onset of COVID-19 [18]. In our dataset, 34.57% of the unscheduled visits to the CEC-CD occurred in the first 2 months after the last episode of COVID-19. This figure can be explained by the persistence of symptoms (in particular dyspnea and fatigue) that negatively influence the quality of life after SARS-CoV-2 infection, thus causing patients to refer to the CEC-CD for medical evaluation. This interval was also shorter for patients vaccinated before the last COVID-19 outbreak compared to those not vaccinated (3.17 vs. 3.90, *p* = 0.0001). Also, 1.09% of the subjects presented at the CEC-CD 13–15 months after the diagnosis of the viral infection with SARS-CoV-2. During this interval, all of these patients had at least one episode of symptoms specific to SARS-CoV-2 infection, but they did not perform a diagnostic test. This result is consistent with the data from the literature that claims that post-COVID symptoms tend to decrease progressively in the first year after the infection [45,46,47].

Residual lung lesions may be correlated with functional involvement and, at least in some patients, with the respiratory symptoms reported in long COVID [48]. A prospective study described a temporary improvement in pulmonary physiology for the majority of patients; however, 24% of patients who had a severe form of COVID-19 without the requirement for mechanical ventilation did not fully resolve lung radiological changes at 12 months after discharge [49]. Our results are concordant with these findings: 44.42% of the patients had abnormal chest imaging findings, and 50.24% of them also reported respiratory complaints at presentation.

Fatigue (51.0%), respiratory (60.4%), and cognitive (35.4%) symptoms are often alleged by patients with long COVID [30,50,51]. Meta-analysis revealed that a significant proportion of individuals have persistent fatigue and/or cognitive impairment following the resolution of acute COVID-19 [52,53]. Patients with dyspnea or fatigue prior to COVID-19 were not selected for this study in an attempt to avoid bias caused by chronic fatigue syndrome [54]. General complaints (e.g., physical asthenia—65.86%) were more frequent, and neurological manifestations (e.g., paresthesia, concentration, and memory disorders—22.54%) were rarer in our dataset compared to the values reported in the literature. Respiratory manifestations (e.g., dyspnea, coughing) were reported by 44.42% of the subjects from our dataset, a value that is in the range of values reported in the literature (dyspnea in post-COVID—36.0–74.3%) [55,56]. Fatigue is not only the most common symptom of COVID-19 but also a persistent one [34,57,58]. A retrospective survey study developed between 1 October and 31 December 2020 reported that fatigue was the main persistent symptom (36%) and was several times more frequent than dyspnea (10.3%) in Romanian patients who had COVID-19 [59]. In our study, 29.54% of the subjects reported general complaints more than 3 months after the onset of COVID-19, and most of them (65.92%) were not vaccinated. Different inclusion criteria may contribute to the higher frequency of general symptoms estimated in our study.

Metabolic dysfunctions seem to be a risk factor for both severe acute and post-acute COVID-19 sequelae [60]. The relationship between type 2 diabetes mellitus and COVID-19 seems bidirectional [61,62]. In the present study, the most common conditions identified after the detection of SARS-CoV-2 infection were impaired fasting glucose, impaired glucose tolerance, and diabetes mellitus (107 cases); 33 of these cases had no other comorbidities.

It has been estimated that the risk of long COVID was up to 42% in unvaccinated individuals and decreased in those who have received a single vaccine dose (30%), two vaccine doses (17%), or a booster vaccine (16%) [63,64,65]. The complete vaccination schedule was reported by 48.14% of subjects from our database. The time interval between the second dose of vaccine to infection with SARS-CoV-2 was 14–180 days. We observed that the age of the subjects (51.5 vs. 56.03 years old, *p* = 0.001) and the time interval from infection to an evaluation in the CEC-CD (3.17 vs. 3.90 months, *p* = 0.0001) were lower for these individuals compared to the values identified for unvaccinated subjects.

## 5. Conclusions

The post-COVID symptoms are heterogeneous and persistent. Age, vaccination status, and the severity of COVID-19 were associated with different characteristics of patients with post-COVID symptoms with unscheduled visits to the CEC-CD. The symptoms for which the patients presented to this medical center—fatigue, dyspnea, and coughing—have caused a long-term (months) quality of life impairment. This aspect can have an important social impact since many of these patients are active people who were absent from work or had a low yield of the activities carried out. The medical evaluation of these patients with long COVID-19 revealed the new onset of certain chronic pathologies after SARS-CoV-2 infection: hypertension, diabetes mellitus, and atopy. A significant percentage of vaccinated people presented a mild form ofda COVID-19. As a result, residual lung damages were identified in significantly lower numbers in vaccinated patients compared to unvaccinated ones.

## Figures and Tables

**Table 1:** Characteristics of subjects involved in the study.

Characteristics	Number of Subjects
Female/male	274/183
Vaccinated	220
Symptoms at presentation to the CEC-CD	
General symptoms (physical asthenia, fatigue)	301
Respiratory symptoms (dyspnea, coughing)	203
Cardiovascular involvement (palpitations, angora, and oscillatory blood pressure)	108
Neurological symptoms (paraesthesia, concentration, and memory disorders)	103
Diabetes mellitus or oral glucose tolerance test (OGTT) (newly identified)	107
Hypertension (newly identified)	50
Hepatocytolysis	33
Pulmonary outstanding involvement	203
Comorbidities	244
Atopies	112
Residual inflammatory biological syndrome	82
Myocarditis/pericarditis post-COVID-19	49
Treatment for COVID-19 (symptomatic/antiviral and symptomatic)	364

**Table 2:** Age differences between subjects, stratified according to different characteristics.

Characteristics Compared.	Age (Years); *p*
Comorbidities (yes vs. no)	61.68 vs. 44.88; <0.0001
COVID-19 severity (moderate or severe vs. mild form)	61.19 vs. 50.87; <0.0001
Pulmonary outstanding involvement (yes vs. no)	58.77 vs. 49.91; <0.0001
Diabetes mellitus (yes vs. no)	60.06 vs. 51.96; <0.0001
Treatment (antiviral and symptomatic vs. symptomatic only)	60.52 vs. 52.14; <0.0001
Cardiovascular signs and symptoms (yes vs. no)	58.56 vs. 52.39; <0.0001
Vaccinated status (yes vs. no)	51.5 vs. 56.03; <0.001
Respiratory symptoms (yes vs. no)	56.11 vs. 52.04; <0.003

**Table 3:** Significant differences between patients with moderate or severe forms and those with a mild form of COVID-19.

Characteristics (Present vs. Absent)	Forms of COVID-19 (Moderate or Severe vs. Mild); *p*
Pulmonary involvement	107 (81.06%) vs. 96 (29.54%); <0.0001
Treatments ^a^	71 (53.79%) vs. 22 (6.77%); <0.0001
Comorbidities	102 (77.27%) vs. 142 (43.69%); <0.0001
Cardiovascular sign and symptoms ^b^	50 (37.88%) vs. 58 (17.85%); <0.0001
General symptoms	105 (79.55%) vs. 196 (60.31%); <0.0001
Atopies	18 (13.64%) vs. 94 (28.92%); <0.0001
Myocarditis	24 (18.18%) vs. 25 (7.69%); <0.001

Notes: ^a^ antiviral and symptomatic treatment vs. symptomatic only treatment; ^b^ palpitations, angora, oscillatory blood pressure values.

**Table 4:** Time interval between the last SARS-CoV-2 infection and admission to CEC-CD.

Characteristics Compared	Time Interval (Months); *p*
Vaccinated (yes vs. no)	3.17 vs. 3.90; <0.0001
Hypertension present (yes vs. no)	4.89 vs. 3.39; <0.0002
Form of COVID (moderate/severe vs. mild)	4.12 vs. 3.32; <0.0003
Treatment (antiviral and symptomatic vs. symptomatic)	4.23 vs. 3.37; <0.0006

## Data Availability

Data supporting the reported results are available from the authors.
